# The Brilliance–Belonging Model: How Cultural Beliefs About Intellectual Ability Undermine Educational Equity

**DOI:** 10.1007/s10648-025-10034-2

**Published:** 2025-06-25

**Authors:** Christina A. Bauer, Aashna Poddar, Eddie Brummelman, Andrei Cimpian

**Affiliations:** 1 University of Vienna, Vienna, Austria; 2 New York University, New York, NY USA; 3 University of Amsterdam, Amsterdam, The Netherlands

**Keywords:** Belonging, Field-specific ability beliefs, FABs, Brilliance stereotypes, Brilliance beliefs, Anxiety, Self-efficacy, Intrinsic motivation, Discrimination, Bias

## Abstract

As societies worldwide grapple with substantial educational inequities, understanding their underlying causes remains a priority. Here, we introduce the Brilliance–Belonging Model, a novel theoretical framework that illuminates how cultural beliefs about exceptional intellectual ability create inequities through their impact on students’ sense of belonging. The model identifies two types of widespread cultural beliefs about ability: field-specific ability beliefs (FABs) and brilliance stereotypes. FABs are cultural beliefs about the extent to which success in an educational context requires exceptional intellectual ability or “brilliance” (e.g., math more so than language). In contrast, brilliance stereotypes are cultural beliefs that associate exceptional intellectual ability with some groups more than others (e.g., individuals from high vs. low socioeconomic status backgrounds). According to the Brilliance–Belonging Model, students from groups targeted by negative brilliance stereotypes are perceived—by themselves and others—as not belonging in contexts where brilliance-oriented FABs are common. These perceptions compromise students’ psychological safety and lead to disempowering treatment by others, resulting in persistent gaps in achievement and representation. Such effects are amplified by the competitive climates to which brilliance-oriented FABs give rise, where pressure to demonstrate intellectual superiority creates particular challenges for students from intellectually stigmatized groups, who often value cooperation over competition. By revealing how cultural beliefs about intellectual ability shape educational outcomes through their effects on belonging, the Brilliance–Belonging Model provides a roadmap for interventions aimed at fostering a sustained sense of belonging among diverse students.

Across the world, educational systems continue to produce stark inequities. Women remain underrepresented in fields such as mathematics, physics, and philosophy, making up less than 25% of full professors in these disciplines at US universities (National Science Foundation, 2023) and similar proportions across European institutions (European Commission, 2021). Students from lower socioeconomic backgrounds face systematic barriers to academic achievement and advancement, from elementary school through higher education (e.g., Arnold & Doctoroff, 2003; Goudeau et al., 2025; Morgan et al., 2022; OECD, 2023). Those from historically marginalized racial and ethnic groups continue to be underrepresented in many academic domains and face persistent achievement gaps (e.g., Ewert et al., 2014; Reardon & Portilla, 2016). These inequities emerge early in students’ academic careers and often widen over time, shaping not only their educational trajectories but also their career opportunities and life outcomes (e.g., Alexander et al., 2007; Chetty et al., 2020).

Understanding the causes of these persistent inequities is crucial for developing effective solutions. While structural barriers such as access to resources and quality education undoubtedly play important roles, a growing body of work also points to cultural beliefs about intellectual ability—particularly beliefs about exceptional intelligence or “brilliance”—as a factor. These *brilliance beliefs* help explain broad patterns of inequity across academia along gender, socioeconomic, and racial/ethnic lines. They shed light on why inequities are systematically more pronounced in some fields (e.g., mathematics, philosophy) compared to others (e.g., biology, education; Cimpian & Leslie, 2017; Leslie et al., 2015). They may also explain inequities *within* fields (e.g., why some math courses show greater inequities than others). To illuminate how brilliance beliefs shape educational outcomes, here we introduce the Brilliance–Belonging Model. This novel theoretical framework reveals how cultural beliefs about exceptional intellectual ability contribute to educational inequities by undermining certain students’ sense that they belong in certain educational contexts.

Research distinguishes between two types of brilliance beliefs that work in concert to undermine certain students’ achievement and representation. First, *field-specific ability beliefs* (FABs) capture people’s views about what it takes to succeed in different academic fields (for a review, see Muradoglu et al., 2023). In fields such as mathematics or philosophy, FABs often emphasize the necessity of exceptional intellectual ability. Second, *brilliance stereotypes* reflect widespread societal assumptions about who possesses such exceptional ability, with brilliance being more strongly associated with men, people from higher socioeconomic status (SES) backgrounds, and White people. These two beliefs operate jointly: When a field is perceived to require brilliance (brilliance-oriented FABs) and brilliance is stereotypically associated with certain groups, students from other backgrounds—women, people from lower-SES backgrounds, and people of color—will face inequitable barriers to their success.

The Brilliance–Belonging Model explains *how* brilliance-oriented FABs and brilliance stereotypes combine to create educational inequities. When a field is believed to require brilliance for success, students from groups that face negative brilliance stereotypes (such as women, people from lower-SES backgrounds, and people of color) are likely to feel—and be treated as though—they do not belong. This perceived lack of belonging sets off a chain reaction: Students may feel psychologically unsafe in these environments, while others may treat them in ways that undermine their potential. These mutually reinforcing experiences of psychological threat and disempowering treatment can ultimately lead to lower achievement and representation in these fields.

Before elaborating on these processes, it is important to be precise about how we conceptualize belonging in this model. Here, we define belonging as the extent to which students are perceived—by themselves as well as others—to fit in with and to be valued in a given educational environment, and the accompanying psychological sense of connection and acceptance that these perceptions foster (e.g., Allen et al., 2021; Cohen, 2022; Walton & Cohen, 2007; Goodenow, 1993; Leary & Baumeister, 1995): When someone belongs in a given environment, they understand at a cognitive level that they are respected by others and are capable of succeeding academically, and they also experience the emotional security and comfort that comes from the perception of being “at home” in that environment.^1^ Hence, this definition of belonging encompasses both the academic and the social facets of belonging (e.g., Allen et al., 2021; Walton & Cohen, 2007).

Overall, the present argument extends prior work in four ways. First, we distill the large amount of previous research on brilliance beliefs into a coherent mechanistic model—the Brilliance–Belonging Model. This model offers novel predictions about how brilliance beliefs create inequities through their effects on belonging in academic environments (see the later section titled *Open Research Questions Highlighted by the Brilliance–Belonging Model*). Second, the model illuminates how multiple forms of inequity—along gender, socioeconomic, and racial/ethnic lines—emerge from common underlying mechanisms involving brilliance beliefs and belonging. Third, the model bridges two previously disconnected research traditions: research on brilliance beliefs, which has underexplored belonging as a critical mechanism, and research on belonging in educational contexts (see Fong et al., 2024, for a recent review), which has extensively documented the psychological significance of belonging but has not fully examined how brilliance beliefs may trigger belonging concerns. Fourth, by identifying some of the psychological mechanisms underlying educational inequities, the present work identifies promising interventions that can contribute to educational equity by countering brilliance beliefs and, as a result, fostering students’ sense of belonging in academic environments.

## Overview of the Article

Our review has three main sections. First, we introduce the two types of brilliance beliefs—field-specific ability beliefs (FABs) and brilliance stereotypes—that create and perpetuate educational inequities through their joint effects. Second, we introduce the Brilliance–Belonging Model, which reveals how these brilliance beliefs create educational inequities by undermining certain students’ sense of belonging. The model specifies both how belonging is threatened by brilliance beliefs and how this threat manifests in lower achievement and representation. Third, we conclude by outlining important open questions that can guide future research on the Brilliance–Belonging Model and intervention approaches that target both brilliance beliefs and belonging concerns.

## Field-Specific Ability Beliefs and Brilliance Stereotypes: Definitions and Evidence

Field-specific ability beliefs (FABs) and brilliance stereotypes are two distinct but related cultural beliefs that shape academic and career trajectories. FABs are beliefs about what qualities are required for success in different fields or contexts, while brilliance stereotypes are beliefs about which social groups possess those qualities. Together, these beliefs can create barriers for certain groups in academic and professional settings.

### What Are FABs?

FABs are cultural beliefs about the “ingredients” needed for success in a particular context (e.g., domain, field, course). At one end of the FAB continuum, *effort-*oriented FABs hold that hard work and persistence are sufficient for success. At the other end, *brilliance-*oriented FABs hold that success *also* requires exceptional intellectual ability—not just hard work (Leslie et al., 2015; Muradoglu et al., 2023). Importantly, brilliance-oriented FABs about a context do not maintain that brilliance is the *only* factor that matters—rather, the idea is that hard work alone is *not enough* for success and must be supplemented by some degree of exceptional intellectual ability.

For instance, US students in grades 1–4 believe that being “super smart” is more important for success in math than in reading, writing, or spelling (Jenifer et al., 2023; Muradoglu et al., 2025; see also Gunderson et al., 2017). This belief pattern extends beyond young students: US academics express more nuanced but similarly aligned views, seeing scholarly success in mathematics and science as more dependent on exceptional intellectual ability than success in language-based disciplines and many other humanities fields (Leslie et al., 2015; Muradoglu et al., 2022). These beliefs have been documented across multiple groups, including high school students (Heyder et al., 2021; Ito & McPherson, 2018) and university students (Storage et al., 2016), as well as the general US population (Meyer et al., 2015).

This concordance across groups illustrates a key feature of FABs: They are consensual within a culture. Even though meaningful individual differences in endorsement exist (e.g., Arnold et al., 2023; Jenifer et al., 2023; Muradoglu et al., 2025), these beliefs are widely agreed upon by members of a culture—in short, they are “in the air.” The evidence also suggests that FABs extend beyond any single cultural context. Beliefs similar to those measured in the USA have been documented in Canada (Maranges et al., 2023), Germany (Asbury et al., 2023; Heyder et al., 2020, 2021), Singapore (Chua, 2021), and Switzerland (Deiglmayr et al., 2019).

FABs are acquired early in development and mirror those of the broader culture by early elementary school (Gunderson et al., 2017; Jenifer et al., 2023; Muradoglu et al., 2025). This early acquisition likely amplifies their influence—these beliefs have extensive opportunity to shape students’ perceptions of belonging in various domains and, consequently, their long-term academic and career trajectories.

The Brilliance–Belonging Model remains agnostic on two key points. First, it makes no claims about whether exceptional intellectual ability is *actually* necessary for success in any given context. People act on their *beliefs*, regardless of whether these beliefs reflect reality. As long as people believe brilliance-oriented FABs to be true, and brilliance stereotypes remain prevalent, these beliefs are likely to create educational inequities.

Second, the Brilliance–Belonging Model remains agnostic about the nature of brilliance itself. Students reason about brilliance, and intellectual ability more generally, through the lens of their intuitive beliefs. For example, students who endorse growth (vs. fixed) mindsets might view brilliance as malleable (vs. stable; e.g., Dweck, 2006), whereas those who hold universal (vs. non-universal) beliefs might consider brilliance attainable by everyone (vs. only some people; e.g., Rattan et al., 2012).^2^ Research suggests that FABs, fixed/growth mindsets, and universal/non-universal beliefs are psychometrically distinct constructs among both children and adults (Asbury et al., 2023; Limeri et al., 2023; Muradoglu et al., 2025), each accounting for unique variance in motivational and achievement outcomes (Asbury et al., 2023; Limeri et al., 2023; Maranges et al., 2023; Muradoglu et al., 2025; Porter & Cimpian, 2023).

The Brilliance–Belonging Model posits that the combination of brilliance-oriented FABs and brilliance stereotypes undermines perceived belonging among intellectually stigmatized students, regardless of their or others’ intuitive beliefs about brilliance. Emerging evidence supports this assumption (Bian et al., 2018a, 2018b; Limeri et al., 2023; Muradoglu et al., 2025; Porter & Cimpian, 2023). However, important questions remain about potential moderation effects. For instance, negative effects might be amplified among students who view brilliance as fixed (vs. malleable) and believe only some people (vs. nearly everyone) can attain it. These questions await future research on the interactive effects between brilliance beliefs and other beliefs about intellectual abilities.

### What Are Brilliance Stereotypes?

While FABs are about the qualities believed to be required for success in different contexts, brilliance stereotypes concern which social groups are thought to possess these qualities. Similar to FABs, brilliance stereotypes are cultural beliefs about exceptional intellectual abilities, but they focus on *perceived group differences* rather than field requirements.

Similar to other stereotypes, brilliance stereotypes have little grounding in reality (e.g., Bian & Cimpian, 2017; Hammond & Cimpian, 2017)—there is no solid evidence for greater brilliance potential among some social groups than others. Indeed, students may face negative brilliance stereotypes even when they possess exceptional intellectual ability. Throughout this article, we use the term “intellectually stigmatized” to refer to individuals from social groups that face such negative stereotypes about their intellectual abilities—specifically women, people from lower-SES backgrounds, and people from historically marginalized racial/ethnic groups.

Evidence collected through various methodologies, including implicit association tests and natural language processing techniques, suggests that—similar to FABs—brilliance stereotypes are consensual within a culture. Specifically, brilliance is commonly associated more strongly with men/boys, with people from higher-SES backgrounds, and with White people than with women/girls, people from lower-SES backgrounds, or people of color (e.g., Bian et al., 2017; Boutyline et al., 2023; Brummelman & Sedikides, 2023; Durante & Fiske, 2017; Fiske et al., 2002; Furnham et al., 2002; Gálvez et al., 2019; Musto, 2019; Rivera & Tilcsik, 2019; Zhao et al., 2022b). Also paralleling FABs, these brilliance stereotypes appear consistent across cultures. Similar patterns have been detected in large cross-national samples (Napp & Breda, 2022; Storage et al., 2020), as well as in focused studies from China (Shu et al., 2022),^3^ Germany (Bauer & Hannover, 2021), Japan (Okanda et al., 2022), Singapore (Zhao et al., 2022b), South Korea (Kim et al., 2024), and the USA (e.g., Bian et al., 2017; Storage et al., 2020).

Like FABs, brilliance stereotypes emerge early in development. Children often internalize their culture’s brilliance stereotypes by elementary school. For instance, by age 6, children perceive gender differences in brilliance: In samples from two different regions of the USA, 6- and 7-year-old girls, but not 5-year-old girls, were less likely than same-age boys to view their own gender as “really, really smart” (i.e., brilliant; Bian et al., 2017; Jaxon et al., 2019; see also Bian et al., 2018a, 2018b). Similar gender-brilliance associations favoring boys and men have been found among 7-year-olds in South Korea and Japan (Kim et al., 2024; Okanda et al., 2022).

Children as young as 6 years of age also perceive those from low-SES backgrounds as less smart than those from high-SES backgrounds (Désert et al., 2009; Mistry et al., 2015; Woods et al., 2005; for an overview, see Brummelman & Sedikides, 2023). This pattern has been documented in both US and French samples.

Although there is less developmental work on racial/ethnic stereotypes about brilliance, related stereotypes about ability in various academic domains seem to emerge in late elementary or even middle school, particularly among racial/ethnic majority groups. For example, White fourth graders (ages 9–10) in the USA viewed Black (vs. White) children as performing worse in math, science, and reading (Rowley et al., 2007), and in academics more generally (Copping et al., 2013). Evidence about stereotype endorsement among Black children is mixed. Some studies indicate they internalize these stereotypes later than White children (specifically, in grades 6–8; Evans et al., 2011; Okeke et al., 2009; Rowley et al., 2007), while others found no endorsement across any tested grades (Copping et al., 2013). Notably, while students from historically marginalized racial/ethnic groups may be less likely to *endorse* negative stereotypes about their ingroup, they often become *aware* of such stereotypes at younger ages than racial/ethnic majority groups (McKown & Weinstein, 2003).

#### Intersectionality in Brilliance Stereotypes

While each person holds multiple social identities—for example, gender, SES, and racial/ethnic identities—most research on brilliance stereotypes has examined these identities in isolation. Yet, holding multiple identities (e.g., being a Black woman) creates unique lived experiences that cannot be predicted by simply adding together the effects of individual identities (Crenshaw, 1991). This recognition has led to widespread calls for intersectional research approaches across the social sciences (e.g., Bowleg, 2008; Cole, 2009; Coles & Pasek, 2020; Collins & Bilge, 2016; Crenshaw, 1991; Harris & Patton, 2019; Lei et al., 2023; McCormick-Huhn et al., 2019; Purdie-Vaughns & Eibach, 2008). 

Two theoretical perspectives offer competing predictions about how multiple stigmatized identities might shape a person’s experiences with brilliance stereotypes. The *double jeopardy model* (e.g., Almquist, 1975; Beal, 2008; Epstein, 1973) suggests that individuals with multiple subordinate identities (e.g., a Black woman) face more prejudice than those with a single subordinate identity (e.g., a White woman), as they encounter discrimination based on each identity depending on the context. In contrast, the *intersectional invisibility model* (Purdie-Vaughns & Eibach, 2008) proposes that people with multiple subordinate identities—who are not seen as prototypical of their social groups (e.g., Black women, when the prototypical Black person is a Black man and the prototypical woman is a White woman; Ghavami & Peplau, 2013)—may experience unique advantages and disadvantages compared to more prototypical group members. Importantly, this model does not predict that multiple stigmatized identities always lead to greater oppression.

Existing research examining brilliance stereotypes at the intersection of gender and race reveals complex patterns that support both theoretical perspectives. On the one hand, there is support for the intersectional invisibility model, in that women of color are sometimes perceived as *more* brilliant than men of color. Specifically, 6-year-old children in the USA view Black women as more brilliant than Black men (Jaxon et al., 2019), while simultaneously viewing White men as more brilliant than White women (Bian et al., 2017; Jaxon, Lei, et al., 2019). This suggests that negative brilliance stereotypes about Black individuals are applied primarily to Black men, while negative stereotypes about women are applied primarily to White women—creating a relative advantage in brilliance perceptions for Black women. Yet, there is also evidence in line with the double jeopardy perspective, where individuals holding multiple negatively stereotyped identities face compounded effects. Specifically, US adults showed weaker implicit associations between Black women and brilliance compared to Black men (Storage et al., 2020).

These complex patterns may reflect the *context-sensitivity* of brilliance stereotypes. In some contexts, people view individuals through the lens of their intersecting identities (e.g., seeing someone specifically as a Black woman). This intersectional perspective can lead to invisibility effects (Purdie-Vaughns & Eibach, 2008), where brilliance stereotypes have reduced impact because they are primarily applied to more prototypical group members (e.g., Black men and White women) rather than to those with intersecting identities. In other contexts, people may view individuals primarily through the lens of a single identity (e.g., focusing on gender alone). When this occurs, judgments about brilliance follow directly from stereotypes about that particular identity. Which perspective people adopt—intersectional or single identity—depends on contextual factors and their goals in the moment (see Petsko et al., 2022). For example, a class on reproductive biology might activate gender as a primary lens, while a US history class on voting rights might activate an intersectional gender × race/ethnicity lens. More broadly, both single-identity and intersectional stereotypes may vary across societies and historical periods, as they are shaped in part by the evolving goals of dominant groups (e.g., White men in the USA) in relation to people with different identities (Lei et al., 2023).

The shifting nature of how people perceive students with multiple intellectually stigmatized identities has crucial implications. Even when intersectional invisibility effects lead to weaker or reversed brilliance stereotypes (e.g., Jaxon et al., 2019), students with multiple stigmatized identities frequently encounter situations where they are viewed through single-identity lenses. Over time, these experiences likely accumulate, and as a result, students who hold multiple stigmatized identities may face more disadvantages in brilliance-oriented contexts than those with fewer or no stigmatized identities. For instance, while Black female students might sometimes benefit from reduced negative stereotyping due to intersectional invisibility, they still regularly encounter situations where they are treated distinctly based on their gender or their race. Consequently, their outcomes in brilliance-oriented contexts—including their sense of belonging—may ultimately be more negative than those of students with a single stigmatized identity. We will explore this possibility further when discussing findings about the intersectional effects of brilliance beliefs.

## The Brilliance–Belonging Model

**Fig. 1:**
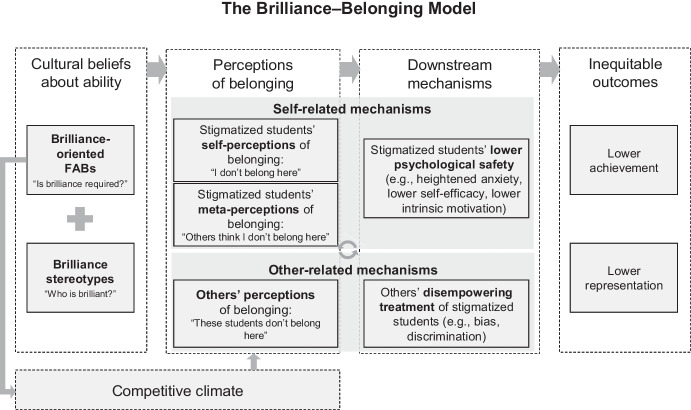
A visual depiction of the Brilliance–Belonging Model that outlines the processes by which brilliance-oriented FABs and brilliance stereotypes undermine the educational outcomes of students from social groups that are stigmatized with respect to their intellectual abilities. FABs = field-specific ability beliefs

The Brilliance–Belonging Model posits that brilliance-oriented FABs and brilliance stereotypes shape educational outcomes through two main pathways: a *self-related* pathway and an *other-related* pathway (see Fig. 1). In the pages that follow, we begin with a high-level overview of the model and then explain how it builds on and extends prior theoretical work. After establishing these conceptual foundations, we turn to a detailed account of the model’s specific predictions and the empirical evidence supporting them.

### Overview of the Core Components of the Model

#### Self-Related Pathway

The self-related pathway encompasses mechanisms *internal* to intellectually stigmatized students. Based on brilliance stereotypes, stigmatized students may believe they do not belong in certain brilliance-oriented contexts (i.e., self-perceptions of low belonging) and may believe others think they do not belong (i.e., meta-perceptions of low belonging). These self- and meta-perceptions of low belonging often undermine psychological safety, increasing anxiety, decreasing self-efficacy, and diminishing intrinsic motivation.

#### Other-Related Pathway

The other-related pathway encompasses mechanisms *external* to intellectually stigmatized students. Relevant others (e.g., instructors, peers) may perceive a poor fit between intellectually stigmatized students and brilliance-oriented contexts. These low-belonging perceptions may lead relevant others to discriminate or show bias against intellectually stigmatized students.

#### Interactive Nature of the Pathways

These pathways mutually reinforce each other. For instance, experiencing disempowering treatment (other-related pathway) might lower a student’s self- and meta-perceptions of belonging (self-related pathway). The resulting effects (e.g., lower achievement, pursuing other subjects) might then reinforce pre-existing brilliance stereotypes, creating a self-perpetuating cycle. Consequently, self-, meta-, and others’ perceptions of belonging may become increasingly aligned over time.

#### Role of Perceived Behavioral Mismatches and Competitive Climates

The Brilliance–Belonging Model identifies two distinct types of perceived mismatches that can undermine intellectually stigmatized students’ sense of belonging in brilliance-oriented contexts. The first, which has been our focus so far, is a perceived *ability* mismatch—the belief that one’s intellectual abilities do not align with the presumed requirements of the field. The second is a perceived *behavioral* mismatch—the sense that one’s typical ways of engaging and interacting do not fit the social norms of the environment.

Behavioral mismatches become particularly salient in the competitive climates that characterize brilliance-oriented contexts (see Fig. 1): The belief that success requires brilliance tends to foster environments where students frequently attempt to demonstrate their intellectual ability publicly (Vial et al., 2022; see also Porter & Cimpian, 2023). Common behaviors might include dominating class discussions, challenging others’ ideas aggressively, or engaging in intellectual one-upmanship. These competitive climates prove especially challenging for intellectually stigmatized students, who may find such performative displays of brilliance uncomfortable or incongruent with their values (e.g., Goudeau et al., 2025). This discomfort with the prevailing behavioral norms is likely to reinforce both types of perceived mismatches: Intellectually stigmatized students, as well as their peers and instructors, may interpret these students’ discomfort with competitive behaviors as further evidence that (a) they lack the requisite intellectual abilities (perceived ability mismatch) and (b) they are unaware of or unwilling to follow the social norms of the environment (perceived behavioral mismatch). Thus, competitive climates create a self-reinforcing cycle that deepens perceptions of not belonging—both students’ own and others’.

### Theoretical Foundations and Extensions

The Brilliance–Belonging Model builds upon and extends several major theoretical traditions in psychology and education. Because these frameworks provide critical insight into the psychological processes that underlie the model’s key pathways, we introduce them here to contextualize the downstream mechanisms through which brilliance beliefs shape student outcomes. Specifically, we examine how the model connects to and extends (a) expectancy-value theory, (b) self-determination theory, and (c) social identity threat theory, each of which helps illuminate the psychological mechanisms through which brilliance beliefs affect students. We then discuss how our model extends (d) person-environment fit frameworks by emphasizing the role of cultural beliefs in shaping perceived belonging. Throughout this section, we highlight how the Brilliance–Belonging Model both draws from and advances these foundational theories.

#### Connections to Expectancy-Value Theory

Expectancy-value theory (e.g., Eccles & Wigfield, 2020; Wigfield & Eccles, 2000) posits that individuals’ beliefs about their likelihood of succeeding in an activity and the value they assign to that activity shape their motivation: Students are more likely to engage and persist in activities that they expect to do well in and that they value.

In brilliance-oriented contexts, intellectually stigmatized students may develop lower expectations for success, not because of actual ability differences but because cultural messaging suggests they do not belong in these contexts as they lack the requisite “brilliance” (e.g., Wang & Degol, 2013). By undermining perceptions of belonging, brilliance-oriented contexts may also undermine the perceived value of pursuing these activities for stigmatized students. Expectancy-value theory distinguishes between different types of values, all of which may be affected in brilliance-oriented contexts. For instance, intrinsic value (enjoyment of an activity) may suffer if brilliance-oriented contexts foster a competitive, exclusionary environment. Utility value (the perceived usefulness of an activity for current or future goals) may also be affected if brilliance-oriented contexts undermine stigmatized students’ sense of belonging and make them question whether the corresponding activity aligns with their broader aspirations. Moreover, perceptions of cost (the negative aspect of value, including the effort, tradeoffs, or emotional toll of engaging in an activity; Perez et al., 2014) may also be heightened for stigmatized students in brilliance-oriented contexts. Potential costs include not only the emotional toll of confronting stereotypes but also the opportunity costs of pursuing brilliance-oriented activities over alternatives that students might (be made to) feel are better aligned with their strengths and aspirations (Matthews & Wigfield, 2024).The Brilliance–Belonging Model extends expectancy-value theory in two key ways. First, while expectancy-value theory acknowledges the role of sociocultural factors in shaping individuals’ expectancies for success and task values (Eccles & Wigfield, 2020), our model identifies a set of cultural messages—namely, brilliance beliefs—that have not previously been integrated into this theoretical framework. Second, our model introduces belonging as a crucial mediator within expectancy-value theorizing: Brilliance beliefs shape students’ sense of belonging, which then influences their expectancies and values.

#### Connections to Self-Determination Theory

The processes described by the Brilliance–Fit Model align with and extend self-determination theory (e.g., Ryan & Deci, 2000). According to this framework, optimal motivation emerges when students’ basic psychological needs—for example, their need to feel connected to others—are satisfied. 

In brilliance-oriented contexts, intellectually stigmatized students are particularly likely to experience threats to these psychological needs. For instance, their self- and meta-perceptions of low belonging may directly compromise their sense of connection or relatedness: Feeling that one does not belong—or that others view one as not belonging—makes it difficult to form meaningful social connections with peers and instructors. In addition, others’ perceptions that stigmatized students do not belong, shaped by brilliance stereotypes, may further reinforce feelings of exclusion. For example, teachers or peers who implicitly assume that certain students lack the ability needed may offer less encouragement, fewer opportunities for collaboration, or lower-quality feedback, all of which can deepen stigmatized students’ sense of disconnection.^4^ When basic psychological needs such as that for relatedness are undermined in this way, intrinsic motivation—the genuine enjoyment and engagement with learning—is likely to suffer (e.g., Deci et al., 1991). Over time, this erosion of motivation can contribute to decreased achievement and persistence in brilliance-oriented contexts, particularly for students from groups targeted by negative brilliance stereotypes.

The Brilliance–Belonging Model extends self-determination theory in two key ways. First, it highlights brilliance beliefs as a cultural factor that shapes the extent to which students’ psychological needs are fulfilled. By identifying brilliance beliefs as a contextual influence, the model helps explain why students from intellectually stigmatized groups experience greater threats to their basic needs in some academic contexts compared to others. Second, the model specifies the psychological mechanisms—namely, self-, meta-, and others’ perceptions of belonging—through which brilliance-oriented environments systematically threaten certain groups’ basic psychological needs. This integration of sociocultural context and psychological process helps illuminate why persistent demographic gaps are particularly pronounced in contexts that emphasize exceptional intellectual talent (e.g., Cimpian & Leslie, 2017; Leslie et al., 2015).

#### Connections to Social Identity Threat Theory

Theories of social identity threat (e.g., Shapiro & Neuberg, 2007; Steele & Aronson, 1995; Thoman et al., 2013) illuminate how concerns about being devalued based on one’s social group membership can undermine performance and persistence (see also Graham et al., 2022; López et al., 2025). These theories explain that when students feel their social identities are devalued in a setting, they experience heightened vigilance and physiological stress responses that can interfere with learning and performance (e.g., Cook et al., 2012; Murphy et al., 2007).

In brilliance-oriented contexts, social identity threat may be particularly acute because stereotypes about brilliance exist that target women, people from lower-SES backgrounds, and people of color. The mere suggestion that a field requires brilliance can trigger concerns about confirming negative stereotypes—concerns that can apply both to oneself as an individual and to one’s group as a whole (as distinguished in Shapiro’s Multi-Threat Framework; Shapiro & Neuberg, 2007)—leading to underperformance and disengagement (e.g., Good et al., 2012; Woodcock et al., 2012). Moreover, social identity threat can create a sense of belonging uncertainty that makes normal academic challenges feel more threatening and diagnostic of one’s fit in the field (Walton & Cohen, 2007).

The Brilliance–Belonging Model advances social identity threat theory by identifying a context’s emphasis on brilliance as a distinct and previously underexamined trigger of identity threat that operates across multiple stigmatized groups simultaneously. The fact that a field’s mere emphasis on brilliance can trigger threat responses shows how seemingly neutral messages about what it takes to succeed in a field can function as powerful sources of identity threat.

#### Connections to Person-Environment Fit Research

Person-environment fit models (van Vianen, 2018; see also Aday et al., 2024; Bauer & Hannover, 2021; Cheryan & Plaut, 2010; Eagly & Karau, 2002; Heilman, 2012; Niedenthal et al., 1985) suggest that individuals’ fit with a given setting determines their achievement. These models typically define fit as the match between a person’s characteristics and a setting’s characteristics. Our model extends this tradition by examining how cultural beliefs about intellectual abilities shape *perceived* belonging/fit, regardless of *actual* abilities. This helps explain why an individual might be perceived as not belonging in a brilliance-oriented context even when their intellectual ability is an objective match to the context.

The following sections detail the self- and other-related pathways of the Brilliance–Belonging Model, followed by discussion of the competitive climates arising from brilliance-oriented FABs. Finally, we examine key individual- and context-level moderators: intersectionality and situational brilliance cues.

### Self-Related Pathway

According to the Brilliance–Belonging Model, the self- and meta-perceptions of low belonging triggered by the combination of brilliance-oriented FABs and brilliance stereotypes activate several related processes that reduce psychological safety: They heighten anxiety, lower self-efficacy, and lower intrinsic motivation among intellectually stigmatized students (see Fig. 1).^5^ While we discuss each process separately for convenience, they are interconnected (e.g., anxiety undermines self-efficacy and vice versa; Muradoglu et al., 2022; Rouxel, 1999).

#### Anxiety

The self- and meta-perceptions of low belonging experienced by intellectually stigmatized students in brilliance-oriented contexts can give rise to anxiety—feelings of apprehension, stress, or concern that relate negatively to students’ academic achievement (Barroso et al., 2021; Caviola et al., 2022). 

Women contemplating hypothetical jobs and educational opportunities that emphasize brilliance as necessary for success experience heightened anxiety (Bian et al., 2018a, 2018b). Their anxiety is specifically prompted by self-perceptions of low belonging—their perception that they are dissimilar to others in brilliance-oriented contexts and lack what is needed to succeed. In a study of university students from the USA, the UK, and Germany, students from low-SES backgrounds experienced more anxiety than their peers from higher-SES backgrounds when they imagined attending a brilliance-oriented (vs. an effort-oriented) university (Bauer et al., 2023; see also Canning et al., 2020). The anticipated anxiety among students from low-SES backgrounds was partially explained by their tendency to think of themselves as relatively less intellectually talented (i.e., a self-perception of low belonging).

Impostor feelings (Clance & Imes, 1978)—a form of anxiety prompted by the perception that one’s competence is lower than needed for success in an educational or professional context—provide additional evidence for these relationships. Women academics in more brilliance-oriented fields experience more impostor feelings than those in less brilliance-oriented fields, while men’s impostor feelings are unrelated to their field’s brilliance-emphasis (Muradoglu et al., 2022).

#### Self-Efficacy

The self- and meta-perceptions of low belonging experienced by intellectually stigmatized students in brilliance-oriented contexts can undermine these students’ confidence in their ability to succeed in respective contexts—that is, their self-efficacy (Bandura, 1997; Eccles & Wigfield, 2002), which is a key predictor of motivation and achievement in school (Schunk & DiBenedetto, 2021).

A sample of US girls from the first and second grades reported lower self-efficacy than boys in math to the extent that they endorsed brilliance-oriented FABs about this domain (Jenifer et al., 2023). Similar gender differences emerged in experimental studies with adults: Women exhibited lower self-efficacy than men in brilliance-oriented contexts (Bian et al., 2018a, 2018b).

With respect to socioeconomic status, low-SES university students exhibited less self-efficacy (measured indirectly by their preference for easier problems when given a choice) compared to their higher-SES counterparts in a brilliance-oriented context but not in an effort-oriented context (Bauer et al., 2023). The SES difference in self-efficacy in the brilliance-oriented context was explained in part by self-perceptions: low-SES students were significantly less likely to see themselves as “talented” and “gifted,” which may signal self-perceptions of low belonging.

#### Intrinsic Motivation

The self- and meta-perceptions of low belonging experienced by intellectually stigmatized students in brilliance-oriented contexts can undermine these students’ intrinsic motivation—the form of motivation that leads students to engage with school activities for the inherent satisfaction, pleasure, or interest these activities provide (e.g., Deci & Ryan, 1985; Wang & Eccles, 2012). As discussed in the previous section on self-determination theory, intrinsic motivation is optimal for well-being, persistence, and achievement in school (Ryan & Deci, 2000).

Effects on intrinsic motivation emerge early in development. Girls as young as 6 or 7 years of age from the USA and South Korea showed less interest than boys in an unfamiliar game ostensibly intended for children who “are really, really smart” (Bian et al., 2017; Kim et al., 2024)—an effect that was particularly pronounced among girls who endorsed brilliance stereotypes themselves. These gender differences in interest were absent when the same unfamiliar game was described as being for children who “try really, really hard.”

Similar patterns persist through adolescence and young adulthood. In a sample of US high school students, the more that students perceived a STEM field to be brilliance-oriented, the less interest girls had relative to boys in pursuing that field in the future (Ito & McPherson, 2018). This relationship was stronger among students who had taken more STEM classes already, suggesting that firsthand exposure to brilliance-oriented contexts reinforces girls’ self-perceptions of low belonging. In a sample of German high school students, girls’ endorsement of brilliance-oriented FABs about math predicted lower intrinsic motivation in this domain even after adjusting for their prior achievement (Heyder et al., 2021); this relationship was not observed among boys.

Among adults, women indicated less interest in brilliance-oriented job opportunities compared to men (Bian et al., 2018a, 2018b). This gender difference in intrinsic motivation was explained in part by women’s lower perceived belonging in the brilliance-oriented job (e.g., women reported lower similarity to others doing this job and were less likely to say that they match the job requirements). The gender gap persisted regardless of whether brilliance was described to participants as fixed or malleable, consistent with the Brilliance–Belonging Model’s claim that FABs can undermine equity regardless of how students think about the nature of intellectual ability.

#### Limitations of Prior Evidence

The Brilliance–Belonging Model predicts that self- and meta-perceptions of low belonging have unique, independent effects on the educational outcomes of intellectually stigmatized students. Yet, prior research testing the Brilliance–Belonging Model has not consistently differentiated between these two types of perceptions (see Fig. 1). For example, Bian et al., (2018a, 2018b) measured perceptions of belonging in a global manner that incorporated both self- and meta-perceptions of low belonging. Such a measure prevents a clear comparison of the relative roles of these perceptions in triggering downstream mechanisms. The closest these researchers came to distinguishing between the two types of perceptions was their measure of stereotype threat (e.g., “I worry that people who work at this company will draw conclusions about me based on what they think about my gender”). This measure can reasonably be interpreted as capturing meta-perceptions of belonging; however, it did not explain women’s greater anxiety in brilliance-oriented contexts, suggesting perhaps that self-perceptions may be more influential. Indeed, when Bauer et al. (2023) isolated self-perceptions in their research, they found that these perceptions explained significant variance in outcomes—but because they did not investigate the role of meta-perceptions as a potential parallel process, a direct comparison remains impossible. Future research should develop separate measures and manipulations to disentangle the effects of self- and meta-perceptions of belonging.

 More broadly, the studies reviewed here as support for the Brilliance–Belonging Model pertained predominantly to gender and SES and, with a few exceptions, were conducted in Western, industrialized nations. Future research should test the predictions of the Brilliance–Belonging Model with respect to the self-related pathway among students from historically marginalized racial/ethnic minority backgrounds, as well as examine these relationships in a more diverse array of cultural contexts.

### Other-Related Pathway

We now turn to the *other-related pathway* (see Fig. 1)—the way that others’ perception that intellectually stigmatized students do not belong in brilliance-oriented contexts contributes to inequities. According to the Brilliance–Belonging Model, these perceptions of low belonging trigger biased, disempowering treatment toward intellectually stigmatized students.

Often, others’ biased treatment discourages intellectually stigmatized individuals from entering brilliance-oriented contexts in the first place. Most of the prior research that speaks directly to this point has focused on gender. For example, adults in the USA refer to fewer women than men for jobs that emphasize brilliance, but this difference is not observed for other jobs (Bian et al., 2018a, 2018b). In chess—a non-academic domain where brilliance is prized—US mentors and parents report that girls and young women have lower potential than boys and young men (Arnold et al., 2023). This bias is heightened among mentors and parents who believe brilliance is necessary for success in chess. Such biased treatment from mentors and parents could discourage female youth players from pursuing chess long-term. Peer bias also emerges in brilliance-oriented contexts. When 5- to 7-year-old children from the USA were asked to select teammates for a game ostensibly intended for children who are “really, really smart,” children chose girls as teammates less often than in a separate condition without brilliance-oriented messages about the game (Bian et al., 2018a, 2018b). Beyond the USA, Hannak, Joseph, Larremore, and Cimpian (2023) investigated the career trajectories of over 85,000 academics globally to determine whether biased treatment explains why fewer women join brilliance-oriented fields. Indeed, they found that members of fields with more brilliance-oriented FABs rated their fields as less welcoming to women, and this perception of biased treatment explained a substantial portion (approximately 35%) of the gender gap in entry into these fields.

Similar patterns emerge among students from low-SES and historically marginalized racial/ethnic backgrounds, though the connection to the Brilliance–Belonging Model is more indirect. For instance, even when their test scores are comparable to those of their non-stigmatized peers, students from low-SES and historically marginalized racial/ethnic backgrounds receive fewer nominations for giftedness programs (Grissom & Redding, 2016; McBee, 2006). Additionally, teachers are less likely to select low-SES students for advanced academic “tracks” compared to their high-SES peers, even with equivalent achievement levels (e.g., Batruch et al., 2019, 2023).

Students do not always have a choice of whether to enter or avoid a brilliance-oriented context (e.g., compulsory math classes), making it crucial to understand how others’ biased treatment affects intellectually stigmatized students not just before but also *after* they have entered such a context. Teachers and peers may unfairly exclude these students from opportunities or provide them with less attention and support—signaling that they do not value them as members of the field. In a recent study, German elementary school teachers’ endorsement of brilliance-oriented FABs about math predicted lower intrinsic motivation in this subject among students who struggled academically in their classes (Heyder et al., 2020). Given that students from low-SES and historically marginalized racial/ethnic minority backgrounds are likely to be overrepresented among these students, this finding aligns with the predictions of the Brilliance–Belonging Model.

While Heyder et al. (2020) did not investigate the classroom dynamics that led to this result, the Brilliance–Belonging Model would predict that teachers’ endorsement of brilliance-oriented FABs might lead them to invest less in and have lower expectations for intellectually stigmatized students. Supporting this prediction, Western European teachers provide fewer opportunities for learning and poorer feedback to students from low-SES and historically marginalized racial/ethnic backgrounds. For example, preschool teachers offer fewer opportunities to children from low-SES backgrounds to participate in whole-class discussions compared to children from high-SES backgrounds with similar levels of language skill (Goudeau et al., 2023). Similarly, teachers give lower grades to intellectually stigmatized students, even when their performance is identical to that of their non-stigmatized peers (Doyle et al., 2023; Zanga & De Gioannis, 2023; see also Copur-Gencturk et al., 2020). Even when teachers seem to express positivity toward intellectually stigmatized students, their behavior may send these students subtle disempowering messages. For example, when two students achieve the same outcome, teachers are more likely to lavish the lower-SES student with inflated praise (e.g., “You did incredibly well!”), leading classmates to infer that this student must be more hardworking but less smart (Schoneveld & Brummelman, 2023).

Overall, the other-related pathway represents a self-fulfilling cycle (e.g., Rosenthal & Jacobson, 1968): When others perceive and treat stigmatized students as if they do not belong in brilliance-oriented contexts, these students face increased barriers to success and persistence in these environments. This treatment can manifest through reduced learning opportunities, lower expectations, subtle messages of exclusion, and differential evaluation—all of which make it more challenging for these students to demonstrate their abilities and maintain their presence in these spaces. When students struggle or leave these contexts as a result, it reinforces others’ initial perceptions that these students do not belong, perpetuating the cycle.

#### Limitations of Prior Evidence

Most of the prior evidence for the other-related pathway of the Brilliance–Belonging Model focuses on gender. Evidence regarding SES and racial/ethnic disparities is more indirect. We encourage more research that tests directly whether students from low-SES and historically marginalized racial/ethnic minority backgrounds experience biased treatment in brilliance-oriented contexts. While considerable evidence demonstrates bias against these students, the extent to which this bias connects to brilliance-oriented FABs and brilliance stereotypes remains unclear.

Another key limitation is that no studies have directly measured others’ perceptions of belonging. Although the evidence shows biased treatment of intellectually stigmatized students, the connection between this bias and perceptions of these students’ belonging requires further investigation. The Brilliance–Belonging Model predicts an important role for these perceptions in the emergence of bias. Research is needed to determine whether teachers and peers who perceive certain students as not belonging in brilliance-oriented contexts are more likely to exhibit biased behavior toward them.

Finally, as with the self-related pathway, most evidence for the other-related pathway comes from Western cultural contexts, warranting more studies testing the model’s predictions outside the USA and Western Europe.

### Intersectional Patterns in Intellectually Stigmatized Students’ Belonging

Building on our understanding of how the self- and other-related pathways shape educational outcomes, we now turn to examining how multiple social identities intersect to influence belonging in brilliance-oriented contexts. The two previously mentioned theoretical frameworks help explain how gender, SES, and race/ethnicity might jointly shape individuals’ experiences. The double jeopardy model (e.g., Almquist, 1975; Epstein, 1973) predicts that individuals holding multiple stigmatized identities experience compounded disadvantages—the combined challenges associated with each stigmatized identity. For example, a woman from a low-SES background might encounter both gender-based and class-based barriers to belonging in brilliance-oriented contexts. In contrast, the intersectional invisibility framework (Purdie-Vaughns & Eibach, 2008) suggests that individuals with multiple stigmatized identities may face unique challenges beyond the simple addition of separate disadvantages.

Evidence from three key studies supports the double jeopardy model’s predictions. In a study comparing German and US students’ experiences, Bauer and Job (2024) found that female students from low-SES backgrounds reported the lowest sense of belonging in brilliance-oriented contexts compared to all other groups. Specifically, these students viewed themselves as least intellectually talented. They also experienced heightened anxiety and demonstrated lower self-efficacy, choosing less challenging problems to work on. Similar patterns emerged when examining gender and race/ethnicity intersections: Female academics from historically marginalized racial/ethnic backgrounds in the USA and Germany reported the strongest impostor feelings (Muradoglu et al., 2022) and lowest levels of belonging (Bauer & Hannover, 2021) in brilliance-oriented contexts compared to other groups.

While these findings demonstrate quantitatively greater disadvantages for individuals with multiple stigmatized identities, questions remain about whether these effects reflect simple double jeopardy or more nuanced intersectional processes. A significant limitation of existing research is that it often combines different minoritized racial/ethnic groups into a single category for analysis, potentially masking important differences between groups. For instance, Hispanic women may encounter gender-based brilliance stereotypes (Bailey et al., 2024), while Black women may face distinct stereotype patterns (Jaxon et al., 2019). These varying stereotype patterns could lead to different experiences of belonging and achievement in brilliance-oriented contexts. Additional research examining specific racial/ethnic groups separately is needed to determine when and how the Brilliance–Belonging Model aligns with predictions from the intersectional invisibility or the double jeopardy frameworks.

### Situational Fluctuations in Intellectually Stigmatized Students’ Belonging

While the previous section examined how multiple social identities shape experiences in brilliance-oriented contexts, brilliance-related inequities can also fluctuate based on *immediate situational cues* (see Boucher & Murphy, 2017; Murphy & Taylor, 2011). These variations occur not only between fields but also within them—for example, between different mathematics classes in the same department.

Specific cues that suggest brilliance is valued in a situation can trigger both the self-related and the other-related pathways. Cues that trigger the self-related pathway might include classroom posters featuring only historical “geniuses” from dominant groups, course syllabi emphasizing the need for “mathematical minds,” or assignment instructions praising “talented” problem-solvers. Even seemingly minor incidents—such as a teacher’s offhand comment about “getting it immediately” or peers forming study groups based on perceived ability—could trigger doubts about belonging among intellectually stigmatized students. Indeed, experience sampling research suggests that low-SES students report more concerns about their intellectual abilities than their higher-SES counterparts in classes that emphasize competitiveness (Canning et al., 2020).

The other-related pathway may also be activated by situational cues, though the specific mechanisms remain to be tested. For instance, mathematics teachers might encounter brilliance cues through departmental messaging that emphasizes “identifying gifted students early,” curriculum materials featuring historical “genius” mathematicians, or professional development sessions focused on “cultivating exceptional talent.” Such cues might lead teachers to scrutinize stigmatized students’ performance more critically, provide less encouraging feedback, or distribute challenging problems unequally among students.

Understanding how specific situational factors trigger these pathways represents an important direction for future research. Such work could help identify ways to create more inclusive classroom environments by minimizing cues that activate brilliance-oriented FABs and their associated inequities.

### Adverse Effects of the Competitive Climates Created by Brilliance-Oriented Contexts

Beyond the immediate impact of specific brilliance cues, brilliance-oriented contexts can create broader competitive climates that pose additional challenges for intellectually stigmatized students’ sense of belonging. When FABs emphasize brilliance as necessary for success, they foster an environment where people feel compelled to demonstrate their intellectual abilities. These contexts encourage constant social comparison and competition as students try to prove they possess the requisite brilliance. Rather than focusing on personal growth and development, individuals feel pressure to distinguish themselves through competitive behaviors that showcase their intellectual superiority (Glick et al., 2018; Vial et al., 2022). Such climates often penalize displays of vulnerability, including intellectual humility (Porter & Cimpian, 2023), making it difficult for students to acknowledge gaps in their knowledge or learn from mistakes.

The behavioral norms that accompany competitive climates are harmful to the well-being and achievement of *everyone* who is exposed to these climates (Koc et al., 2021; Regina & Allen, 2023; Vial et al., 2022). For instance, the fact that competitive climates penalize displays of intellectual humility makes it less likely for students—regardless of their social identities—to ask questions and solicit help when needed, both of which are key components of learning (Porter & Cimpian, 2023; see also Limeri et al., 2023; Muradoglu et al., 2025).

However, these climates can be especially uncomfortable for intellectually stigmatized students for two key reasons. First, the constant pressure to prove one’s brilliance in competitive environments may heighten social identity threat for women, individuals from low-SES backgrounds, and racial/ethnic minorities. These repeated demands to demonstrate intellectual ability create numerous opportunities for negative evaluation and confirmation of harmful stereotypes, potentially undermining students’ perceptions of belonging. Second, these climates clash with how many intellectually stigmatized students are socialized. For example, girls are socialized from an early age to behave in ways that are sensitive to others’ needs and well-being rather than putting their own interests first (e.g., Block et al., 2018, 2025). Similarly, individuals from low-SES (vs. high-SES) backgrounds are socialized to be attuned to the needs of others and value relationships over personal gains (e.g., Belmi & Laurin, 2016; Stephens et al., 2012). The communal, other-oriented behavioral norms endorsed by low-SES individuals are in direct opposition to the behavioral norms fostered by the competitive climates to which an emphasis on brilliance gives rise. The same conclusion applies to many racial/ethnic minority students in Western contexts, who endorse communal and cooperative values more strongly than their peers from racial/ethnic majority groups (e.g., Dasgupta et al., 2022; Fryberg & Markus, 2007; Smith et al., 2014). This dual burden—managing both heightened social identity threat and the mismatch between communal values and competitive norms—may lower these students’ own perceptions of belonging and lead their instructors and peers to doubt their belonging as well, triggering the self- and other-related pathways described earlier.

#### Inequitable Outcomes

The Brilliance–Belonging Model suggests that brilliance-oriented FABs and brilliance stereotypes, operating through self- and other-related mechanisms, lead to inequitable outcomes for intellectually stigmatized students: lower achievement and, ultimately, lower representation in brilliance-oriented contexts (see Fig. 1).

Evidence links brilliance-oriented FABs and brilliance stereotypes to the underrepresentation of intellectually stigmatized students at multiple levels of the education system. A survey of 1820 US academics across 30 fields found that fields emphasizing exceptional intellectual ability (e.g., math, philosophy) graduated fewer women and Black PhDs than fields that placed less emphasis on brilliance (e.g., psychology, education; Leslie et al., 2015). These brilliance-oriented FABs predicted inequitable representation patterns beyond other field-level characteristics, such as math-intensiveness (Cimpian & Leslie, 2015), selectivity, or compatibility with work/life balance. Using a different measure of FABs—the frequency of “brilliant” and “genius” in over 14 million anonymous US university instructor reviews on RateMyProfessors.com—Storage et al. (2016) replicated these findings.^6^ This word-count measure predicted women’s and Black students’ underrepresentation at both PhD and bachelor’s degree levels. High school students and the general population also held these beliefs, which predicted women’s underrepresentation among PhD holders (Ito & McPherson, 2018; Meyer et al., 2015).

Hannak, Joseph, Larremore, and Cimpian (2023) examined gender differences in academic career trajectories using ORCID.org profiles of over 85,000 academics worldwide. Women were underrepresented among those entering fields with more brilliance-oriented FABs and overrepresented among those leaving these fields—a pattern consistent across career stages and geographical regions, including the USA, Canada, Europe, Latin America, and Asia.

While direct evidence connecting brilliance-oriented FABs and brilliance stereotypes to lower student achievement remains limited, the Brilliance–Belonging Model suggests this connection through established mechanisms such as self-efficacy and discrimination. Extensive prior evidence indicates that such mechanisms influence student achievement (e.g., Eccles & Wigfield, 2002; Malouff & Thorsteinsson, 2016; Walton & Cohen, 2007). Thus, we expect that intellectually stigmatized students are often unable to achieve their full potential in brilliance-oriented contexts.

To fully understand these educational disparities, it is crucial to examine how brilliance-oriented FABs and brilliance stereotypes interact with broader structural inequities—and how these patterns differ across gender, socioeconomic status, and racial/ethnic lines. Students from low-SES and historically marginalized racial/ethnic backgrounds face disadvantages across educational domains (e.g., Arnold & Doctoroff, 2003; Ewert et al., 2014; Song, 2011; Wilbur & Roscigno, 2016), with brilliance-oriented environments potentially intensifying these challenges. Women’s educational outcomes follow a different pattern—they typically outperform male peers throughout schooling and comprise a majority of university degree holders (DiPrete & Buchmann, 2013; Voyer & Voyer, 2014). Their disadvantages emerge specifically in contexts emphasizing brilliance-oriented FABs (e.g., Hannak et al., 2023; Leslie et al., 2015). These varying patterns highlight how educational outcomes stem from multiple factors beyond brilliance-oriented FABs and brilliance stereotypes, including systemic barriers affecting students from low-SES and historically marginalized racial/ethnic backgrounds—barriers such as residential segregation and school funding disparities.

An important area for future research concerns the intersectional effects of brilliance-oriented FABs and brilliance stereotypes. Individuals with multiple stigmatized identities—such as women of color or students from low-SES, racially marginalized backgrounds—may face compounded belonging threats in brilliance-oriented contexts. As a result, these individuals may experience more severe reductions in psychological safety and heightened exposure to disempowering treatment from others, ultimately leading to larger disparities in achievement and representation compared to peers with a single stigmatized identity. Investigating these intersectional dynamics is essential for understanding how brilliance beliefs contribute to educational inequities and for designing interventions that effectively support students with multiple marginalized identities.

## Discussion and Future Directions

We developed the Brilliance–Belonging Model to shed light on the mechanisms by which brilliance-oriented FABs and brilliance stereotypes undermine equity in education. This model highlights *perceptions of belonging* as a core mechanism: whether students see themselves as belonging in a brilliance-oriented context, whether they believe that others see them as belonging, and whether others actually see them as belonging. If the answer to any of these questions is “no”—a likely prospect for students who are intellectually stigmatized—a cascade of self-reinforcing processes is set into motion that culminates in lower achievement and representation in brilliance-oriented contexts (see Fig. 1).

Notably, the implications of the Brilliance–Belonging Model extend well beyond educational contexts. While educational settings remain crucial—as they often represent the foundation where inequities first emerge and become entrenched—the model’s principles operate across multiple settings. These beliefs shape workplace dynamics, where women face persistent barriers in STEM careers and leadership positions due to assumptions about intellectual ability (e.g., Bian et al., 2018a, 2018b; Hannak et al., 2023; Heck et al., 2021; Muradoglu et al., 2022). Similar patterns emerge in competitive domains traditionally associated with intellectual prowess, such as chess, where gender, socioeconomic status, and race/ethnicity affect participation and achievement (e.g., Arnold et al., 2023). Understanding how brilliance beliefs operate across domains—from professional environments to competitive venues—reveals their pervasive influence on achievement-related choices, persistence, and success throughout individuals’ lives. This broader applicability suggests that interventions targeting brilliance beliefs could have far-reaching effects across multiple sectors where brilliance-oriented thinking creates barriers for intellectually stigmatized groups.

This final section addresses two critical questions for future research and practice. First, we examine the socialization and intergenerational transmission of brilliance-oriented FABs and brilliance stereotypes. Second, we analyze potential intervention strategies to disrupt their cascading effects. We conclude the section by highlighting key open research questions highlighted by the Brilliance–Belonging Model. These questions are intended to guide future research in this area.

### Socialization and Transmission of FABs and Brilliance Stereotypes

Research examining the developmental origins of brilliance beliefs has primarily focused on documenting when, rather than *why*, these beliefs emerge. Gender-based brilliance stereotypes appear early in development (e.g., Bian et al., 2015, 2018), as do brilliance stereotypes based on socioeconomic status (e.g., Désert et al., 2009; Woods et al., 2005) and race/ethnicity (e.g., Copping et al., 2013; Rowley et al., 2007). Similarly, field-specific ability beliefs emerge early in children’s academic trajectories (e.g., Jenifer et al., 2023; Muradoglu et al., 2025). However, the socialization experiences underlying these developmental patterns remain poorly understood.

Parents and teachers, who themselves hold brilliance stereotypes and brilliance-oriented FABs about subjects such as mathematics (Brummelman et al., 2023; Heyder et al., 2020; Musto, 2019; Zhao et al., 2022b), likely play a key role in transmitting these beliefs. For instance, parents’ gender-brilliance stereotypes correlate with their children’s (Zhao et al., 2022b). Yet, the specific mechanisms of transmission require investigation. Do explicit conversations about ability shape children’s beliefs, or do more subtle influences drive this process—such as parents differentially attributing sons’ (vs. daughters’) achievements to ability more than effort (Jacobs & Eccles, 1992; Yee & Eccles, 1988)? Beyond family influences, children encounter cultural messages through television, movies, and books that may reinforce brilliance beliefs (e.g., Boutyline et al., 2023; Elmore & Luna-Lucero, 2017; Gálvez et al., 2019). For example, do television programs present more men and boys, more individuals from high-SES backgrounds, and more individuals from racial/ethnic majority groups in roles that are thought to require brilliance (e.g., scientists, inventors)?

The classroom environment presents another crucial context for investigating the transmission of brilliance beliefs. Which classroom features and teacher practices signal a focus on brilliance? How do classrooms and teachers convey to students that success in some fields or domains ostensibly requires brilliance, whereas success in other fields or domains does not? One relevant source of information may be educational materials such as textbooks. For example, textbooks may explicitly link success in some domains to high levels of intellectual ability (e.g., describing famous physicists, mathematicians, and philosophers as geniuses who did not have to work hard to achieve their success). Another source may be teachers’ language. For example, teachers may explicitly link success on some tasks to high levels of intellectual ability. They may praise students who excel in mathematics as “smart” but those who excel in reading as “hardworking.” Even when the difference between these types of praise is subtle and seemingly benign, children readily pick up on its meaning (Brummelman & Dweck, 2020; Cimpian et al., 2007; Mueller & Dweck, 1998), regardless of whether they are praised themselves or overhear a classmate being praised (Zhao et al., 2020, 2022a). For example, when 5-year-old children overhear another child being praised for not having to work hard to succeed, they devalue effort (Zhao et al., 2022a). Over time, children may infer that success in some fields requires more brilliance than success in others. We encourage research that tests these classroom-based transmission processes directly.

Understanding these transmission mechanisms serves two crucial purposes. First, it illuminates how brilliance beliefs perpetuate across generations. Second, it shifts the focus from individual students to systemic factors. Current interventions often target intellectually stigmatized students directly, aiming to boost their confidence or their growth mindsets. While well-intentioned, this approach risks placing an undue burden on these students and suggesting to policymakers that educational inequities can be addressed through individual-level psychological interventions alone (Brummelman & Sedikides, 2023; Brummelman & Ziemer, 2023). More effective approaches might target classroom, school, or broader educational system features to address the root causes of brilliance-oriented FABs and brilliance stereotypes. This aligns with efforts to create “growth mindset cultures” in classrooms (Hecht et al., 2023; Murphy et al., 2021) and highlights the importance of carefully considering intervention strategies based on the Brilliance–Belonging Model.

### Intervention Approaches Rooted in the Brilliance–Belonging Model

The Brilliance–Belonging Model points to different possible intervention approaches to disrupt the negative effects of brilliance beliefs. Below, we first discuss intervention approaches that may appear promising but face significant limitations. We then propose alternative approaches that are better supported by evidence.

#### What Might not Work

The two sets of beliefs central to the Brilliance–Belonging Model are brilliance-oriented FABs and brilliance stereotypes. In theory, counteracting either belief should disrupt the mechanisms outlined in the model. If brilliance-oriented FABs are counteracted so that success in a context is no longer tied to brilliance, then brilliance stereotypes should become irrelevant to the perceived belonging of intellectually stigmatized students. Conversely, if brilliance stereotypes are counteracted so that students’ social identities are no longer used as a proxy for their intellectual potential, then brilliance-oriented FABs should be less likely to undermine the perceived belonging of entire groups of students.

While directly targeting brilliance stereotypes might seem intuitive, the effectiveness of such interventions faces significant challenges. Although stereotypes can change over time (e.g., Charlesworth & Banaji, 2019; Eagly et al., 2020), they rarely show robust, lasting change in response to targeted intervention (e.g., Lai et al., 2016). This resilience is unsurprising given that these beliefs are embedded in, and reinforced by, broader cultural contexts (e.g., Boutyline et al., 2023; Elmore & Luna-Lucero, 2017; Gálvez et al., 2019). Additionally, the language used to challenge stereotypes can have unintended consequences (e.g., Cimpian et al., 2012). For example, statements such as “Girls can be just as brilliant as boys!” may inadvertently reinforce the idea that boys represent the standard for brilliance (e.g., Chestnut et al., 2021). Also notable, attempts to make brilliance more inclusive by broadly labeling groups and individuals as “brilliant” may inadvertently reinforce rather than challenge the problematic notion that brilliance is required for success and thus further undermine intellectually stigmatized students’ perceived belonging.

Another common approach—exposing intellectually stigmatized students to successful ingroup role models—has important limitations. Role models are only inspiring when their success seems attainable; if their achievements seem out of reach, role models can actually *lower* students’ perceived belonging (for a review, see Gladstone & Cimpian, 2021). In addition, if role models try to encourage ingroup members by presenting themselves as brilliant, they may face social backlash (e.g., Rudman & Glick, 2001) and may also inadvertently reinforce the perception that the status quo in their respective fields is fair and meritocratic (Verniers et al., 2024). They may also be dismissed as exceptions that prove the rule, while simultaneously perpetuating the notion that brilliance is necessary for success.

#### What Might Work

Given these considerations, we recommend interventions targeting the FAB component of the Brilliance–Belonging Model. The goal would be to reduce brilliance-oriented FABs and thereby increase perceptions of stigmatized students’ belonging, potentially improving their long-term outcomes.

To illustrate what a FAB-focused intervention might look like, consider a hypothetical early intervention program targeting elementary school children’s beliefs about math success. Such an intervention might use engaging stories and activities that would allow children and parents to explore together different “recipes for success” in mathematics that do not rely on brilliance. These recipes could emphasize strategies such as developing problem-solving tools, cultivating curiosity, working effectively with others, and building a positive relationship with mathematics (see Schaeffer et al., 2018, for a related app-based intervention approach targeting math anxiety). By presenting success as achievable through various pathways other than brilliance, such an intervention could help decouple the notion of math achievement from brilliance.

However, several factors are crucial for effective implementation. First, interventions might be most effective if they are delivered by authority figures in brilliance-oriented contexts (e.g., teachers) rather than through direct-to-student computer programs alone. Instructors’ status as content experts adds credibility and serves as an “institutional signal” that these alternative beliefs are the norm, which is often an effective vehicle for change (e.g., Tankard & Paluck, 2016).

Second, interventions should avoid directly refuting brilliance-oriented FABs, as this approach is often ineffective (Yeager et al., 2016) and can backfire by making such beliefs seem common and reasonable. Instead, interventions should present alternative frameworks for understanding success without explicitly challenging existing beliefs.

Third, these alternatives need to be more than simple “effort matters” messages. The literature has focused heavily on the brilliance end of the FAB spectrum, with alternatives often labeled simply as “effort-oriented FABs” (e.g., Hannak et al., 2023). However, claiming that effort alone determines success may not be persuasive and could inadvertently blame intellectually stigmatized students for their difficulties. Instead, interventions should present a comprehensive, scientifically grounded account of how people actually achieve success in a domain. This should draw on research about effective cognitive and metacognitive processes (e.g., Benjamin, 2007; Ericsson et al., 1993; Kuhn, 2022), motivational factors (e.g., Duckworth & Gross, 2014; Dweck, 2006; Eccles & Wigfield, 2002; Elliot & McGregor, 2001), and necessary material and social supports (e.g., Brummelman, 2020; Granovetter, 1973; Jackson, 2020; Pomerantz et al., 2007). A key challenge for the scientific community is to translate this evidence into accessible messages that provide compelling alternatives to brilliance-oriented FABs.

### Open Research Questions Highlighted by the Brilliance–Belonging Model

In addition to synthesizing the literature on FABs and brilliance stereotypes into a systematic account, the Brilliance–Belonging Model is generative: Throughout, we pointed out gaps in the literature and formulated new research questions that would be fruitful to test. We have gathered these questions in Table 1, alongside a few novel questions that we have not articulated so far.

**Table 1:** Open research questions generated from the Brilliance–Belonging Model

**Field-specific ability beliefs (FABs) and brilliance stereotypes**
**1. How do field-specific patterns of brilliance orientation emerge and persist?** - Why are brilliance-oriented FABs stronger in some fields than others? - What maintains these differences across fields over time?
**2. How do brilliance stereotypes develop and become culturally entrenched?** - What cultural, social, and psychological factors contribute to the early emergence of brilliance stereotypes? - Why do similar brilliance stereotypes appear across different cultures and societies? - How do societal power structures and historical inequities shape the content and targets of brilliance stereotypes?
**3. Do different beliefs about intellectual ability moderate the effects of brilliance-oriented contexts on belonging?** - Do students’ mindsets about intelligence (fixed vs. growth) moderate the impact of brilliance-oriented FABs on belonging? - Do beliefs about the universality of the potential for brilliance moderate the impact of brilliance-oriented FABs on belonging? - Do different combinations of beliefs about brilliance (e.g., fixed and non-universal vs. malleable and universal) lead to different patterns of vulnerability in brilliance-oriented contexts?
**Self-related pathway**
**4. What are the distinct roles of self- and meta-perceptions of belonging?** - How do they independently contribute to educational outcomes? - How do they interact with each other over time?
**5. How does the self-related pathway operate across different cultural contexts and racial/ethnic groups?** - Do self-perceptions of belonging manifest differently across racial/ethnic groups within Western societies? - How do self-perceptions of belonging operate in non-Western cultural contexts? - How do historical and societal differences in educational systems affect how the self-related pathway functions?
**6. How do different combinations of belonging perceptions interact to shape student outcomes?** - How do misalignments between self-, meta-, and others’ perceptions of belonging affect student experiences (e.g., when a student feels they belong but believes others doubt their belonging)? - What factors predict alignment versus misalignment between different types of belonging perceptions? - How do different patterns of belonging perceptions evolve over time? - Which types or combinations of belonging perceptions are most predictive of student outcomes?
**Other-related pathway**
**7. How does the other-related pathway operate for students from low-SES and historically marginalized racial/ethnic backgrounds?** - How directly does bias against these groups connect to brilliance-oriented FABs? - What are the specific mechanisms through which this bias manifests in educational settings?
**8. Do the perceptions of teachers, parents, and peers affect students in brilliance-oriented contexts *differentially*?** **Are some social actors’ perceptions more impactful than others, and how might these asymmetries inform targeted interventions?** - When these perceptions conflict, which have greater influence on students’ educational outcomes? - How might the relative influence of different social actors’ perceptions change across development or educational transitions?
**Competitive climates**
**9. How do competitive climates emerge and operate in brilliance-oriented contexts?** - What specific behaviors and interaction patterns characterize these competitive climates? - How do others’ competitive displays of intellectual ability affect intellectually stigmatized students’ self- and meta-perceptions of belonging? - Do competitive climates have a different impact on students from groups with different cultural values about competition versus cooperation?
**Situational activation of FABs**
**10. How do situational cues activate and reinforce brilliance-oriented FABs?** - What specific elements (e.g., posters of “geniuses,” emphasis on quick understanding, course materials) trigger brilliance-oriented FABs? - How do different ways of framing success and ability in moment-to-moment classroom interactions activate brilliance-oriented FABs? - How do repeated exposures to brilliance cues accumulate to strengthen brilliance-oriented FABs over time?
**Intersectionality**
**11. How do specific combinations of stigmatized identities shape experiences in brilliance-oriented contexts?** - How do intersectional effects vary across different academic domains?
**Cultural transmission**
**12. Through what specific mechanisms do parents transmit brilliance beliefs to children?** - Do explicit conversations shape children’s beliefs? - How do subtle influences (such as differential attribution patterns) contribute?
**13. How do popular media representations shape brilliance beliefs?** - How do television shows, movies, and social media portray intellectual ability across demographic groups? - What implicit messages about brilliance do children’s entertainment and books convey?
**14. How do everyday classroom interactions and practices transmit messages about the role of brilliance in academic success?** - How do different forms of teacher feedback shape students’ beliefs about brilliance? - How do assessment methods and grading practices communicate messages about the role of brilliance in success? - How do peer grouping strategies and classroom discussion patterns reinforce or challenge brilliance-oriented FABs?
**Intervention approaches**
**15. What alternative frameworks for understanding success could effectively replace brilliance-oriented FABs?** - How can research on effective learning processes be translated into accessible messages? - How can these alternatives be communicated without inadvertently reinforcing brilliance beliefs?
**16. What systemic interventions could effectively counter brilliance beliefs?** - How can educational systems be restructured to promote more inclusive definitions of success?
**17. How might interventions that reframe rather than reduce brilliance-oriented FABs affect student belonging?** - How do different ways of defining brilliance (e.g., in terms of behaviors versus traits) affect stigmatized students’ belonging? - Could expanding cultural definitions of brilliance to include collaborative behaviors reduce inequities? - What are the tradeoffs between redefining versus de-emphasizing brilliance in intervention approaches?

## Conclusion

How can we foster greater equity in educational outcomes? Our review underscores the critical impediments posed by brilliance-oriented FABs and brilliance stereotypes. The Brilliance–Belonging Model highlights how these beliefs threaten intellectually stigmatized students’ belonging across educational contexts through the interplay of self- and other-related mechanisms. When a field emphasizes brilliance as necessary for success, students from groups targeted by negative brilliance stereotypes face a double challenge: They doubt their own belonging while simultaneously confronting others’ doubts about their belonging. These challenges are often amplified by competitive climates that arise in brilliance-oriented contexts. The implications of the Brilliance–Belonging Model extend beyond educational settings to any contexts or domains associated with intellectual prowess. Looking ahead, we identify two critical priorities: understanding how these beliefs are socialized and transmitted across generations, and developing evidence-based interventions that present alternative frameworks for understanding success without relying on notions of brilliance or individual effort.

The Brilliance–Belonging Model provides a systematic framework for understanding how cultural beliefs about intellectual ability create and perpetuate educational inequities. By identifying belonging as a crucial mediator between these beliefs and student outcomes, the model offers new insights for intervention approaches while generating important questions for future research. We anticipate that this theoretical framework will catalyze research aimed at fostering a sustained sense of belonging among diverse students and, ultimately, creating more equitable educational outcomes.

## Footnotes

1 This conceptualization differs somewhat from traditional approaches in educational psychology, which often focus on belonging primarily as an enduring emotional connection to school (e.g., Allen et al., 2018; Goodenow, 1993). While our model incorporates these chronic affective elements, it places greater emphasis on belonging as an assessment of one’s fit within specific educational contexts. This expanded view allows us to better capture how brilliance beliefs shape both short-term perceptions of fit and longer-term emotional bonds with educational environments.

2 In earlier theorizing, FABs were measured with items that referenced “an innate gift or talent” and aptitudes that cannot be taught (e.g., Leslie et al., 2015), which blurred the boundary between brilliance-oriented FABs and fixed mindsets. More recent theorizing and measures no longer make assumptions about the nature of brilliance (Muradoglu et al., 2023, 2025; Porter & Cimpian, 2023).

3 Although the general pattern of brilliance stereotypes is consistent across cultures, important exceptions exist. For instance, Shu et al. (2022) found that 5- to 7-year-old children in both China and the USA perceived Asian women as more brilliant than Asian men—a pattern that contradicts the typical gender-brilliance stereotype. This suggests that brilliance stereotypes can be more complex at the intersection of different social identities and may vary depending on cultural context.

4 Although we focus here on threats to students’ needs for relatedness as an illustration, the Brilliance–Belonging Model also has implications for the other basic psychological needs described by self-determination theory. For instance, brilliance beliefs could undermine students’ sense of competence by framing success as dependent on exceptional ability. They could also diminish students’ sense of autonomy if students feel constrained by stereotypes or external judgments about their intellectual potential.

5 In this section, we use terminology that, in our view, best captures the specific psychological processes at the heart of the Brilliance–Belonging Model. However, it is important to note the conceptual overlap with terms used in the theoretical frameworks discussed in the preceding section. For instance, self-efficacy relates closely to expectancy beliefs in expectancy-value theory, while intrinsic motivation parallels the concept of intrinsic value. Similarly, anxiety represents one manifestation of the psychological threat discussed in social identity threat theory. The core mechanisms of the Brilliance–Belonging Model apply across these various theoretical frameworks, even though the specific terminology may vary depending on the theoretical tradition.

6 Why might the mere presence of these words be a proxy for FABs? If the members of a field believe that exceptional intellectual ability is the key to success, they are likely to convey this belief to students, and in turn students may themselves begin to evaluate people in the field (in this case, their instructors) in terms of whether they possess this valued ability.
